# Resistance to dryness of Escherichia coli O157:H7 strains from outbreak in Sakai City, Japan, 1996.

**DOI:** 10.3201/eid0402.980232

**Published:** 1998

**Authors:** Y Iijima, M Matsumoto, K Higuchi, T Furuta, T Honda


8. St George TD. Are there common features in lyssavirus diseases? Arbovirus Research in Australia. In: Uren MF, Blok J, Manderson LH, editors. Proceedings of the 5th symposium of the Commonwealth Scientific and Industrial Research Organization/Queensland Institute of Medical Research (CSIRO/QIMR); 1989; Brisbane, Australia. Brisbane, Australia: CSIRO/QIMR; 1989; p. 264-7.
9. Swanepoel R, Leman PA, Burt FJ, Zachariades NA, Braack TG, Rollin PE, et al. Experimental inoculation of plants and animals with Ebola virus. Emerg Infect Dis 1996;2:321-5.
10. Hill JE, Smith JD. Bats, a natural history. Austin (TX): University of Texas Press; 1984.

### Resistance to Dryness of *Escherichia coli* O157:H7 Strains from Outbreak in Sakai City, Japan, 1996

**To the Editor**: A large outbreak of *Escherichia coli* O157:H7 with more than 6,000 cases occurred in Sakai City, Osaka Prefecture, Japan, in July 1996 (1); after the outbreak, more than 1,000 secondary infections occurred in the families of the patients (2). We studied the resistance of *E. coli* O157:H7 to dryness because the survival on surfaces of inert materials under dry conditions may be related to the transmissibility of the strains.

*E. coli* O157:H7 strains grown on 3.0% nutrient broth with 1.5% agar for 20 to 22 hours at 37°C were suspended at a concentration of approximately $5 \times 10^8$ cfu/ml in a 10% skim milk, 0.5% NaCl solution. Aliquots (10 µl) of bacterial suspensions were spread to approximately 10 $cm^2$ on plastic petri plates for bacterial culture and dried under the air flow of a clean bench until no aliquots were evident. After storage in the dark at room temperature, bacteria were harvested with saline and gauze and the viable number was counted (n = 3).

The log reductions 12 hours after drying were employed to show the resistance levels of *E. coli* O157:H7 to dry stress. The log reductions of the three strains from the Sakai City outbreak (RIMD0509950, RIMD0509894, and RIMD0509951) were 0.04 ± 0.34, 0.14 ± 0.06, and 0.20 ± 0.60, respectively (mean = 0.13), whereas those of the *E. coli* O157:H7 strains from the other cases varied from 0.71 ± 0.18 to 4.57 ± 1.02. (RIMD0509861, 0.71 ± 0.18; ATCC35150, 1.15 ± 0.35; RIMD0509826, 1.23 ± 0.60; RIMD0509742, 1.28 ± 0.18; ATCC43890, 1.31 ± 0.17; RIMD0509933, 1.48 ± 0.19; RIMD0509932, 1.48 ± 0.22; RIMD0509765, 3.60 ± 0.24; RIMD0509764, 4.57 ± 1.02; mean = 1.87). Although the strain from the Sakai City outbreak (RIMD0509950) survived for at least 35 days under the dry conditions, the strains from the other cases had no viable cells after 7 days. The log reductions of *E. coli* JM109 and DH10B strains were more than 10, and no viable cells were detected in 12 hours.

The strains from the Sakai City outbreak also showed marked acid resistance. Acid resistance of *E. coli* O157:H7 has been reported to depend on *rpoS*, which is induced in a stationary phase (3). Since the *E. coli* O157:H7 strains in a stationary phase were more resistant to dry and acid stresses than those in a log phase, *rpoS* may also be associated with resistance to dry stress. However, no deletions of *rpoS* were detected by polymerase chain reaction analysis in the *E. coli* O157:H7 strains used in this experiment. Further study on the mechanism of resistance will be needed to establish new strategies for eradicating the bacteria.

A case-control study by the Ministry of Health and Welfare of Japan showed that uncooked radish sprouts were the vehicle of the largest outbreak in Sakai City (4). In two small outbreaks of *E. coli* O157:H7 in March 1997, the vehicle of infection might have been radish sprouts; therefore, the possible contamination of white radish seeds with *E. coli* O157:H7 has been discussed (5). If such contamination was present, dry resistance might be involved in the survival on or in white radish seeds because the bacteria were exposed to dry conditions for a long period before sprouting. We propose that dry resistance be considered an important factor in infection.

**Yoshio Iijima,*† Mayumi Matsumoto,* Kumiko Higuchi,* Taro Furuta,* and Takeshi Honda†**
*Saraya Biochemical Laboratory, Kashiwara, Japan; †Institute for Microbial Diseases, Osaka University, Suita, Japan

### References

1. National Institute of Health and Infectious Diseases Control Division, Ministry of Health and Welfare of Japan. Verocytotoxin-producing *Escherichia coli* (Enterohemorrhagic *E. coli*) infections, Japan, 1996-June, 1997. Infectious Agent Surveillance Report 1997;18:153-4.
2. Takatorige T. Study on the patients of the diarrheal disease outbreak among schoolchildren in Sakai City; occurrence of infections among the families of the schoolchildren. In: Report of Osaka Prefecture Research Society for Enterohemorrhagic *E. coli* Infectious Disease, Osaka; 1997. p. 85-96. (in Japanese)

3. Lin J, Smith MP, Chapin KC, Baik HS, Bennett GN, Foster JW. Mechanisms of acid resistance in enterohemorrhagic *Escherichia coli*. Appl Environ Microbiol 1996;62:3094-100.
4. Michino H, Araki K, Minami S, Takaya S, Sakai N. Investigation of large-scale outbreak of *Escherichia coli* O157:H7 infection among schoolchildren in Sakai City, 1996. In: Proceedings of the 32nd Joint Conference on Cholera and Related Diarrheal Diseases, U.S.-Japan Cooperative Medical Science Program(USJCMSP); 14-16 Nov 1996; Nagasaki University, Nagasaki, Japan. USJCMSP: 1996. p. 84-9.
5. Nathan R. American seeds suspected in Japanese food poisoning epidemic. Nat Med 1997;3:705-6.

### Irradiation Pasteurization of Solid Foods

**To the Editor:** Osterholm and Potter have made a strong case for irradiation pasteurization of solid foods that enter kitchens as raw agricultural commodities, such as meat, poultry, and seafood (1). Irradiation pasteurization was advocated to protect against foodborne diseases caused by common pathogens such as *Campylobacter*, *Cryptosporidium*, *Escherichia coli*, *Listeria*, *Salmonella,* and *Toxoplasma* (2). An additional rationale for irradiation pasteurization is bacterial resistance to antimicrobial drugs, a major health concern, which will undoubtedly increase in magnitude unless new approaches become available (3). The widespread use of antibiotics in animal husbandry may be the cause of some of this resistance, for example, in vancomycin-resistant enterococci associated with the agricultural use of glycopeptide antibiotics (4,5). Furthermore, resistance to glycopeptide antibitiotics can be transferred from enterococci to other gram-positive organisms, at least in the laboratory (6). Thus, resistant bacterial strains from animal sources may enter the human population through contaminated food without necessarily causing immediate disease but resulting in expanded human reservoirs of antimicrobial resistance through horizontal gene transfer (7). When such bacterial strains are subsequently transmitted to a susceptible person, serious disease could result, which would be exceedingly difficult to treat (8). Irradiation pasteurization of solid foods could reduce the magnitude of transfer of resistance genes through contaminated foods.

**Stephen Moses and Robert C. Brunham**
University of Manitoba, Winnipeg, Canada

### References

1. Osterholm MT, Potter ME. Irradiation pasteurization of solid foods: taking food safety to the next level. Emerg Infect Dis 1997;3:575-6.
2. Monk JD, Beuchat LR, Doyle MP. Irradiation inactivation of food-borne microorganisms. Journal of Food Protection 1995;58:197-208.
3. Gold HS, Moellering RC. Antimicrobial-drug resistance. N Engl J Med 1996;335:1445-53.
4. Bates J, Jordens JZ, Griffiths DT. Farm animals as a putative reservoir for vancomycin-resistant enterococcal infection in man. J Antimicrob Chemother 1994;34:507-14.
5. Van de Bogaard AE, Jensen LB, Stobberingh EE. Vancomycin-resistant enterococci in turkeys and farmers [letter]. N Engl J Med 1997;337:1558-9.
6. Leclerq R, Derlot E, Weber M, Duval J, Courvalin P. Transferable vancomycin and teicoplanin resistance in *Enterococcus faecium*. Antimicrob Agents Chemother 1989;33:10-5.
7. Davies J. Inactivation of antibiotics and the dissemination of resistance genes. Science 1994;264:375-82.
8. Swartz MN. Hospital-acquired infections: diseases with increasingly limited therapies. Proc Natl Acad Sci U S A 1994;91:2420-7.

### Emerging Infectious Diseases in Brazil

**To the Editor:** Hooman Momen's update on emerging infectious diseases in Brazil (1) appears to be based solely on notifiable disease data, which cannot adequately describe the current situation. Additional data in several areas may be useful.

Parasitic diseases: Dr. Momen's update restricts itself to protozoal diseases and does not distinguish between mucocutaneous and visceral leishmaniasis. Visceral leishmaniasis is in fact expanding in many suburban and urban areas in the northeast. Mucocutaneous leishmaniasis, after a small retreat following extensive deforestation, has made a comeback; and in many suburban areas in Rio de Janeiro and São Paulo, in the southeast, transmission is occurring, probably because of changes in sandfly ecology (1).

A helminthic disease of interest is mansoni schistosomiasis, which has been expanding its area of transmission, reaching over to Santa Catarina, in the south, to Pará in the north, expanding also westward, to Mato Grosso and Mato Grosso do Sul. The number of cases, as well as the associated illness, has possibly been reduced, but there is no doubt that the disease can be found in a much larger area than 20 years ago. Other emerging helminthiases of interest, albeit not of public health concern, are

